# Seal or varnish? Overcoming the challenges of conducting a CTIMP in a research naive, primary-school based environment through a risk-adapted approach

**DOI:** 10.1186/1745-6215-14-S1-P18

**Published:** 2013-11-29

**Authors:** Simon Hutchings, Ivor Chestnutt, Barbara Chadwick, Jacqui Nuttall, Rebecca Playle, Kerenza Hood

**Affiliations:** 1 Cardiff University, Cardiff, UK

## 

The Seal or Varnish study is a pragmatic randomised trial to evaluate the relative cost and effectiveness of pit and fissure sealants and fluoride varnish in preventing dental caries in high-risk children in South Wales. In partnership with the Cardiff & Vale UHB Community Dental Service (CDS), the trial utilises the infrastructure of an existing school-based dental programme to conduct clinical assessments/intervention delivery via mobile dental units.

While this research naive setting is novel for a CTIMP, in accordance with the 2012 MRC/DH/MHRA joint guidance for risk-adapted approaches to the management of CTIMPs, the low risk nature of the interventions have allowed justification of several risk-based adaptations to the management and conduct of the trial. In addition, through the use of consultation exercises with a representative parents group, adaptations to the standard NRES templates for participant information sheets/consent forms have also been developed in an attempt to maximise uptake onto the trial.

Employment of the risk-adapted approach has minimised additional administrative burden placed on the CDS in implementing the trial compared with the existing dental programme. The adaptations made to the consent process and participant materials have received positive feedback, however consent rates for the trial were found to be lower than previous years' response rates to the dental programme in the majority of schools.

This trial represents one of the first experiences of employing a risk-adapted approach to management of CTIMPs, highlighting several areas for consideration when designing and implementing trials involving low-risk interventions in novel environments.

